# Taxonomy for the Rehabilitation of Knee Conditions (TRAK), a Digital Intervention to Support the Self-Care Components of Anterior Cruciate Ligament Rehabilitation: Protocol of a Feasibility Study

**DOI:** 10.2196/resprot.6402

**Published:** 2016-12-05

**Authors:** Emma Dunphy, Fiona L Hamilton, Kate Button

**Affiliations:** ^1^ EHealth Unit Primary Care and Population Health University College London London United Kingdom; ^2^ School of Healthcare Sciences Cardiff University Cardiff United Kingdom

**Keywords:** musculoskeletal, ACL, rehabilitation, eHealth, self management

## Abstract

**Background:**

Rupture of the anterior cruciate ligament (ACL) is common, especially in the active population. In defining the problem of ACL rehabilitation, this study draws from the knowledge that improved self-care, strength, and fitness are associated with better outcomes. Traditional rehabilitation involves regular physiotherapy, but it is not clear what the optimal way for delivering rehabilitation is, and it varies widely across the world. Evidence for treatments are discussed in the literature, however standard length of rehabilitation and frequency of appointments are unknown. Additionally, current rehabilitation models in the National Health Service (NHS) struggle with catering to large volumes of patients and the lengthy time span over which rehabilitation is delivered. The use of eHealth (the Internet in health care) has been successful at delivering behavior change to a number of diverse patient groups. In physiotherapy, problems such as exercise compliance, exercise technique, and managing a broad program of rehabilitation and advice can be challenging. An eHealth intervention called Taxonomy for the Rehabilitation of Knee Conditions (TRAK) to support self-management and behavior change has been developed by patients and clinicians, and acceptability studies have yielded positive results. TRAK is not an exercise rehabilitation protocol; it is a tool to support ACL rehabilitation with personalized plans, prompts, and logs to help adherence and videos and instructions to improve quality and address queries. The patients have their own log-ins and can email their physiotherapist through the website. This novel platform is directly in line with current NHS England, National Institute for Health and Care Excellence, and NHS Improvement agendas that call for rehabilitation initiatives using both technology and supported self-management for patients. This study forms part of a research platform to identify a best practice model of ACL care from the literature and opinions of key stakeholders. Patients’ exercise programs and duration of treatment are still based on individual needs, but use of the website may offer improved self-management and function and reduced health resource use.

**Objective:**

This is a feasibility study to establish recruitment, retention, sample size estimates, and practicality of collecting outcome measures to inform a future trial comparing the TRAK intervention, which has been rigorously designed to address the challenges of ACL rehabilitation, to usual care.

**Methods:**

This is a feasibility study comparing 2 groups: standard care and standard care plus eHealth. It will use convergent parallel mixed methods where both qualitative and quantitative data are sought for a more thorough understanding of the objectives. Primary outcomes relate to feasibility, including recruitment, retention, and usage. Secondary outcomes relate to health resource use and patient-rated outcome measures.

**Results:**

This research expects to establish the feasibility of a full-scale randomized controlled trial to explore whether patients who use an eHealth intervention to support ACL rehabilitation have better outcomes plus improved self-efficacy and reduced health resource use than a usual care group.

**Conclusions:**

The study will provide essential information to support the development and powering of a future clinical trial of eHealth and physiotherapy for patients with ACL reconstruction in the NHS.

## Introduction

### Background

Anterior cruciate ligament (ACL) rupture is a relatively common injury among those who are physically active [1-3]. Surgery is still the most common way to manage these patients and rehabilitation is essential [1-5] for “maximizing potential for patients to live a full and active life within their family, social networks, education/training and the workplace where appropriate” [5]. The optimal way for delivering rehabilitation is not clear, and current methods vary widely across the world [6-8]. Evidence for treatments is much discussed in the literature. This study addresses how current methods of service delivery in the National Health Service (NHS) struggle to cater to large volumes of patients and the lengthy time span over which rehabilitation is delivered [9-11]. Costing ACL rehabilitation in the NHS is not currently possible given that no standard guideline of care exists.

Some of the challenges of ACL rehabilitation include patient adherence, quality and type of exercises, motivation, and incomplete rehabilitation [3,4,6-8]. eHealth, defined as the use of the Internet in healthcare [12], could improve ACL rehabilitation when used as a tool to support behavior change and greater self-management [13,14]. To date, the use of eHealth has been successful at delivering behavior change to a number of diverse patient groups [12,15-17].

Taxonomy for the Rehabilitation of Knee Conditions (TRAK) is a website that targets behavior change to support self-management. It provides an individualized exercise program, progress log for key exercises, information section for each stage of rehabilitation, prompts, and videos and instructions to support the quality of rehabilitation [18] (see Multimedia Appendix 1). Patients log in to a personal account and have an embedded email link to the physiotherapist who oversees their program.

The TRAK intervention was developed at Cardiff University and has been the subject of a series of development studies to aid self-management in a cohort of patients with knee problems [10,19,20]. It is based on the TRAK ontology, which models standard care knee rehabilitation [20]. Further studies including an acceptability study of TRAK in an ACL population in the NHS are underway in a London hospital. TRAK is in line with current NHS England, National Institute for Health and Care Excellence (NICE), and NHS Improvement agendas and initiatives around rehabilitation using both increased technology and increased support for patients to self-manage [5,21,22].

### Objectives

A feasibility study is needed to establish recruitment, retention, sample size estimates and the practicality of collecting outcome measures. It will illuminate the mechanisms that may affect a future clinical trial comparing the TRAK intervention, which has been rigorously designed to address the behavioral challenges of ACL rehabilitation, to usual care.

## Methods

### A Mixed Methods Randomized Feasibility Trial

This study will use convergent parallel mixed methods where an embedded qualitative study will help expose the mechanisms influencing feasibility data such as recruitment, retention, and usage [23,24]. An online randomization tool will be used to mitigate imbalance between the arms and to assess whether it is possible to recruit to both arms of the study: standard care and standard care plus TRAK. This study will provide essential information to support the development and powering of a future clinical trial of eHealth and physiotherapy for patients with ACL reconstruction in the NHS.

The study will aim to recruit participants from the ACL rehabilitation pathway at a North London NHS Hospital. It will explore patient acceptance of the randomization process and of the burden of participation in a study such as submitting to demography profiling and outcome collection, attending training sessions and interviews, and committing to use the intervention [25-27].

Collecting information for an economic evaluation as part of research is important for potentially informing policy [25]. The study will assess the feasibility of collecting EQ-5D-5L, a validated outcome measure for health status which can be used for health and economic appraisal. It is used to calculate quality-adjusted life-years (QALYs) in a full trial to appraise health resource use in both arms of the study [28]. Descriptive statistics and data completeness for patient-completed health care resource use questionnaires will be reported. Methods, ease, and data completeness of collecting number and duration of physiotherapy appointments will be a particular focus of the work. This work will be supervised by a clinical trials unit health economist who will inform the feasibility trial procedures, including how data is captured and reported and how to deal with uncertainty in the data. 

A second part of the study will use semistructured interviews with patients and physiotherapists on their experiences of using TRAK. A schedule of questions will be used to provide an in-depth understanding of the user perspective of the intervention and the participation burden of the study that may have implications for a future trial. Conversations will be taped, transcribed verbatim, and analyzed using a thematic analysis discussed below.

### Selection Criteria

All adults immediately post ACL reconstruction who have been referred to the ACL rehabilitation program, are able to read and write English and give written informed consent, and have access to the Internet at home will be considered for this study. Individuals with complex comorbidities or surgeries such as multiligament reconstruction or fracture will be excluded from the study.

### Potential Ethical Considerations

Ethical approval will be sought from the Health Research Authority. There is a risk that using TRAK will not address an individual patient’s needs. The risk of this has been minimized by conducting a series of development research projects leading up to this study.

There is a risk that the participant’s symptoms deteriorate but remote monitoring through email will not identify this quickly. Since face-to-face treatment still applies and the patient can be identified when attending the group, this risk will be minimized. Patients will also be encouraged to air concerns with their therapist via email through the website.

There is a burden of time placed on the patient whereby they have to learn to use the website and take the time to log in daily. However, participants will have access to many aspects of TRAK beyond completion of the study through remote log-in, and using TRAK for some individuals will mean that rehabilitation can fit in better around their lifestyle.

### Consent

Treating clinicians will identify suitable participants when they attend the ACL group during their face-to-face consultation about their knee condition. If the individual is interested in finding out more about the study and gives verbal consent, the research team will be informed. All individuals will then speak to the lead applicant about the study and participation will be explained. The time of the lead applicant has been funded for this activity.

### Sample Size 

Eligible and consenting patients will be recruited from a North London physiotherapy department. Patient recruitment will begin following completion of preliminary work packages on July 1, 2018. There are on average 2 new patients per week in the ACL group based on local audit data, which equates to 60 patients over 30 weeks who may be eligible for recruitment. There is potential for 15 further patients to be recruited from the first phase of rehabilitation bringing the potential recruits to 75 patients [29,30]. As the inclusion criteria are broad, it is expected that most patients will be eligible, and early patient feedback indicates patients will be keen to partake. Recruitment stops after 30 weeks, which allows the last recruited patient 7 months to participate. This is enough time to deliver on the study objectives of measuring feasibility although not necessarily enough time for patients to complete the program [30].

A sample size of 35 in each arm is recommended in feasibility studies in order to provide sufficient data and precision of means and variances to inform a future randomized controlled trial (RCT) [30]. Once patients have given consent, baseline measures will be collected. Each participant will then be randomized to either treatment or standard care group. This will be done through an online randomizing service. All will be given standard induction and then a TRAK induction for the intervention arm.

Retention will be measured by follow-up outcomes at markers of 6 weeks, 3 months, 6 months, and end of care or the trial. Usage of the website to determine uptake will be measured by the frequency of log-ins, email contacts, and attendance at face-to-face sessions. This will inform the retention rate and the engagement of patients with the intervention.

To assess the acceptability of the website as a self-management support tool, the patients in the TRAK arm of the study will not be obliged to attend weekly face-to-face sessions (although a minimum attendance will be stipulated). Attendance becomes a key measure of self-efficacy, where less face time may indicate greater self-efficacy and therefore informs the possibility of reduced face time for eHealth users in a future RCT. 

Patient and public involvement (PPI) feedback that informed this application has indicated it is acceptable to randomize to either arm of the study. However, retention and usage data will show this more accurately and have significant implications on understanding of demand, implementation, and practicality of researching this new intervention in further studies.

### Arms of the Study

#### Treatment as Usual Group

ACL rehabilitation is an exercise group using evidence-based milestones to progress through a mix of strength and neuromuscular control exercises. Stages 1 and 2 are weekly, and stages 3 and 4 are every 2 weeks. Those who consent and are randomized to the usual care group will have a usual induction to the ACL rehab program, including

- Recommended amount of exercise
- Types of exercise (personalised, related to goals)
- Appropriate use of ice, crutches, or bracing as individually indicated
- Education on potential red flags, expected milestones, and challenges of each stage

Patients will progress through the 4 phases of rehabilitation according to their ability. At each face-to-face session they will be assessed by their physiotherapist for quality of movement, strength, and neuromuscular control toward goal achievement.

#### Intervention Group

If randomized to the intervention arm, patients will have initial education on the use of TRAK to support their rehabilitation. They will be offered follow-up teaching sessions if required. Patients will have access to the 5 dimensions of TRAK:

- Contact with the physiotherapist via email
- Expert knowledge base for each stage of the program detailing milestones, common problems, and a summary of the evidence base
- Individualized exercise program chosen by their physiotherapist, including videos and instructions of each session. There is a technique guide that advises the patient how to do the exercises correctly
- Exercise log to record exercise participation, progressions, and measures of leg strength and effort level
- Prompt system to remind patients to adhere

At each face-to-face session patients will be assessed by their physiotherapist for quality of movement, strength, and neuromuscular control and appropriate progressions toward goal achievement. Their exercises will be modified on their TRAK interface to reflect progress. Use of behavior change tools like logs, goal reviews, and use of prompts are recorded with usage data.

### Outcome Measures

Patients will complete outcomes at 6 weeks, 3 months, 6 months, and final visit. This is in line with previous RCTs in the field [6,31-33].

#### Primary Outcomes

- Recruitment
- Retention
- Usage such as log-ins, number of pages visited, response to prompts, and log use will inform influence of behavior change tools
- Cost analysis
  - Face to face time with physiotherapists
  - Number of appointments
  - Consultant visits
  - General practitioner visits
  - Email contacts
  - Physiotherapist time outside of class

#### Secondary Outcomes

Patient-rated outcome measures (PROMs) are chosen as the most likely outcome measures for use in a full RCT, depending on feasibility results.

- Knee Injury and Osteoarthritis Outcome Score [34]
- Health Resource Use Questionnaire [35]
- Stanford Self-Efficacy Questionnaire [36]
- EQ-5D-5L [28]

####  Usage of TRAK Website

- Log-ins
- Pages visited
- Log input
- Strength progressions input
- Strength gains (Limb Symmetry Index and Return to Sport After Injury Scale)
- Email contacts to physiotherapists

### Adverse Events

Adverse events such as infection, reinjury, or failure to progress are rare but will be monitored throughout at face-to-face sessions and via patient-clinician email. Patients will be alerted to these possibilities in their inductions, in the class, and on the TRAK website.

### Methods for Protecting Against Bias

With the help of the clinical trial unit, an online randomizing process will be used for this trial to help control against bias. A physiotherapist who is blinded to treatment allocation will collect PROMs from patients. PROMs by their nature are bias-limiting because they are filled in by patients and not clinicians.

### Data Analysis and Frequency of Analysis

Standard procedures of data collection will be adhered to and guided by practices of the clinical trials unit. The qualitative and quantitative data strands are to be collected and analyzed simultaneously and with equal priority [23].

Quantitative data will be analyzed as follows: binary and other categorical measures will be summarized using frequencies and percentages and continuous measures using means and standard deviations (or medians and interquartile ranges for very skewed distributions). All outcome measures will be summarized separately by study arm. Differences in outcomes between arms will be estimated using multilevel linear or logistic regression models with a random effect of person to account for the repeated measures on individuals over time. Potential therapist effects will be appropriately modelled. The precision of estimates will be assessed using 95% confidence intervals. Power analyses will be conducted to calculate the sample size necessary to detect an effect of the intervention in a future RCT. 

The qualitative analysis will be done using a thematic analysis approach. This sees the data itself, rather than the theory, driving the process. The themes emerging from the interviews are grouped stringently so all the interview data relating to a particular theme are recorded. An explicit process of analysis will be outlined and reported, especially with regard to how data are weighted and reported secondary to frequency of occurrence or explanatory value [37]. The process will be largely inductive and will develop an epistemology of the ACL patient experience where preconceived ideas are suspended in favor of the unique perspectives of the participants. The patient’s “perception, thought, memory, imagination, and emotional experience of an event” is liberated and fully informs our understanding [38]. Interview data will be analyzed by the chief investigator and cross-checked by another supervisor.

Expected outputs:

- Determining the feasibility of an RCT by gathering sufficient data to power a trial as outlined
- Determining the acceptability of TRAK as a tool of behavior change to physiotherapists and patients and illuminating the mechanisms that influence feasibility measures
- Determining the feasibility of gathering costing data to inform an economic analysis alongside a potential RCT

### Patient and Public Involvement

Participant feedback will be sought from the patient steering group at quarterly meetings throughout the trial or by request. Patients were part of the design of TRAK, and they informed the content and filmed the exercise videos. They checked all lay project documents for the ethics process. A National Institute for Health Research PPI panel reviewed the study favorably, and changes were made based on feedback.

## Results

Recruitment to this study will be pending successful funding of the study. Recruitment will aim to begin in July 2018 following completion of preliminary work packages. Ethical approval will be sought through the Health Research Authority in January 2017 pending successful funding of the study. The study will be overseen by a trial steering committee.

## Discussion

Impact of the study will be the delivery of TRAK ready to test in an RCT at the end of the feasibility study. The feasibility of collecting data toward a phase 3 effectiveness and cost-effectiveness study and the future direction of eHealth interventions in the management of ACL patients in the NHS will be established.

## Appendix

Multimedia Appendix 1 Images from the Taxonomy for the Rehabilitation of Knee Conditions website.

## Abbreviations

ACL: anterior cruciate ligament
NHS: National Health Service
NICE: National Institute for Health and Care Excellence
PPI: patient and public involvement
PROM: patient-rated outcome measure
QALY: quality-adjusted life-year
RCT: randomized controlled trial
TRAK: Taxonomy for the Rehabilitation of Knee Conditions
