# Supplementary figures and images for: Taxonomy for the Rehabilitation of Knee Conditions (TRAK), a Digital Intervention to Support the Self-Care Components of Anterior Cruciate Ligament Rehabilitation: Protocol of a Feasibility Study
Source: JMIR Res Protoc. 2016 Dec 5;5(4):e234. doi: 10.2196/resprot.6402 (PMC5168535; doi:10.2196/resprot.6402)

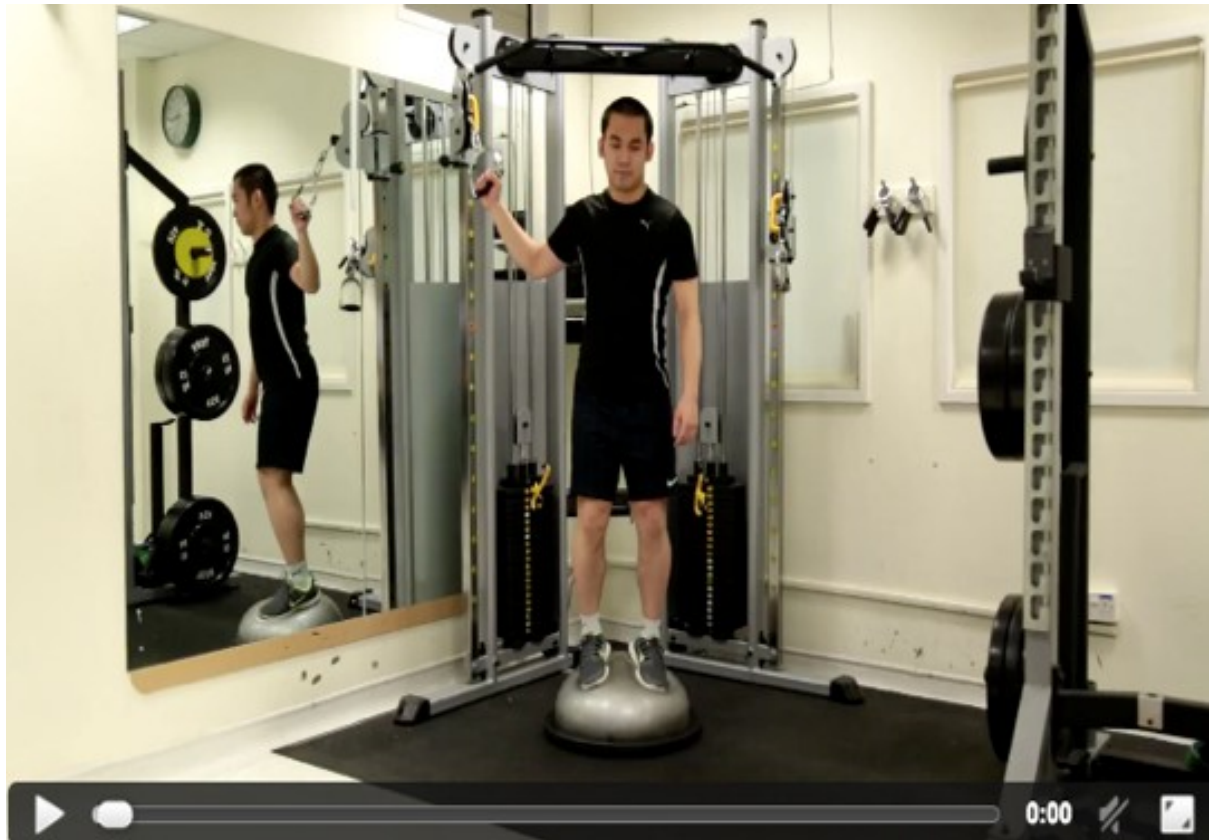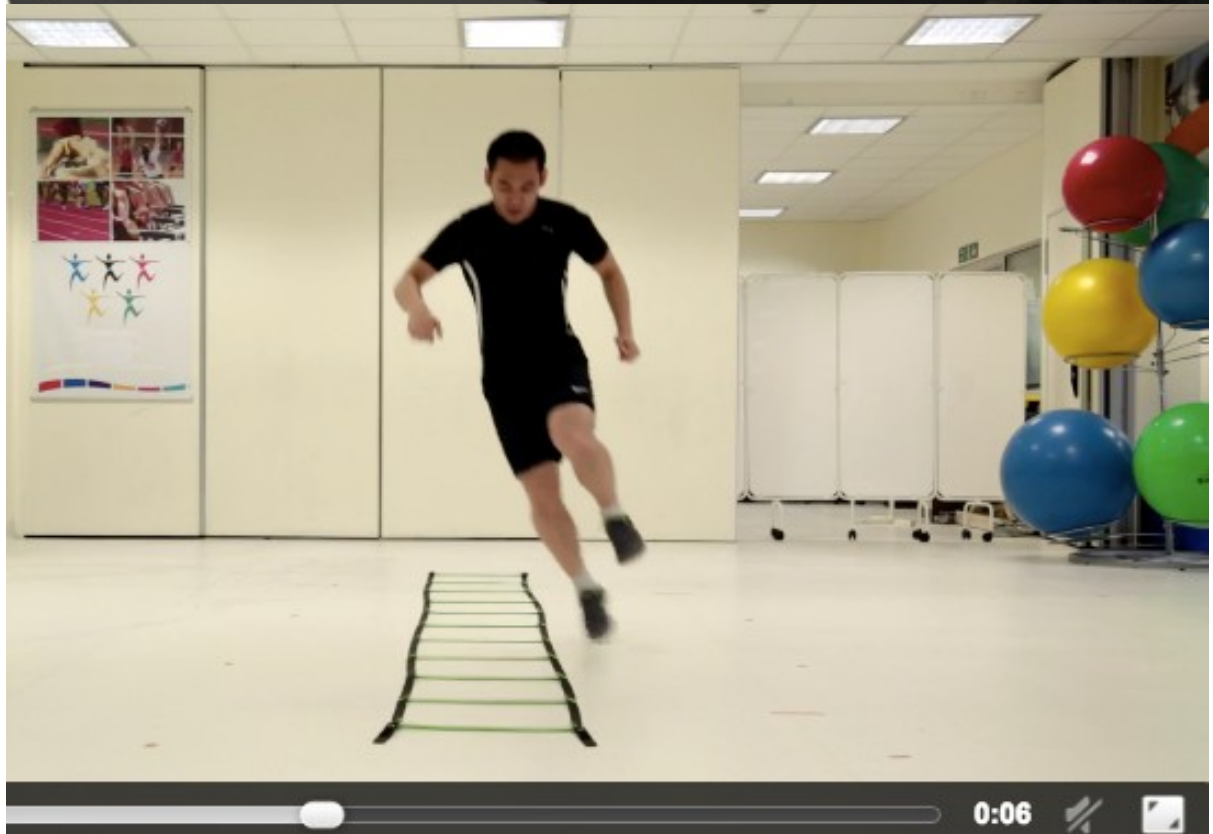

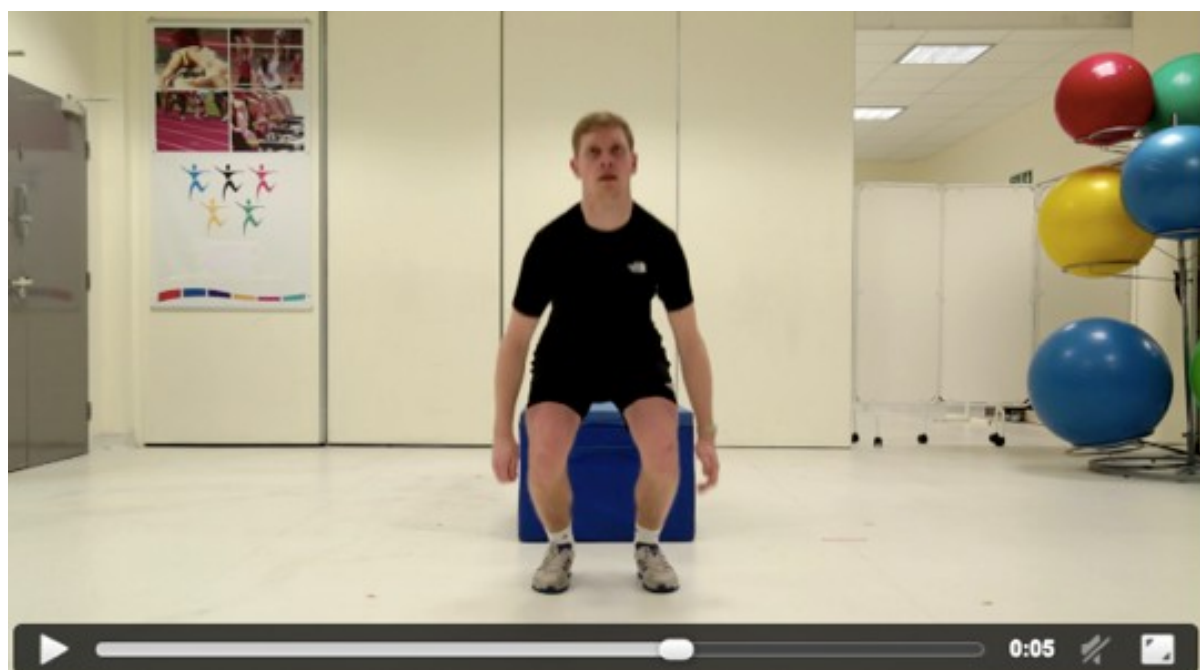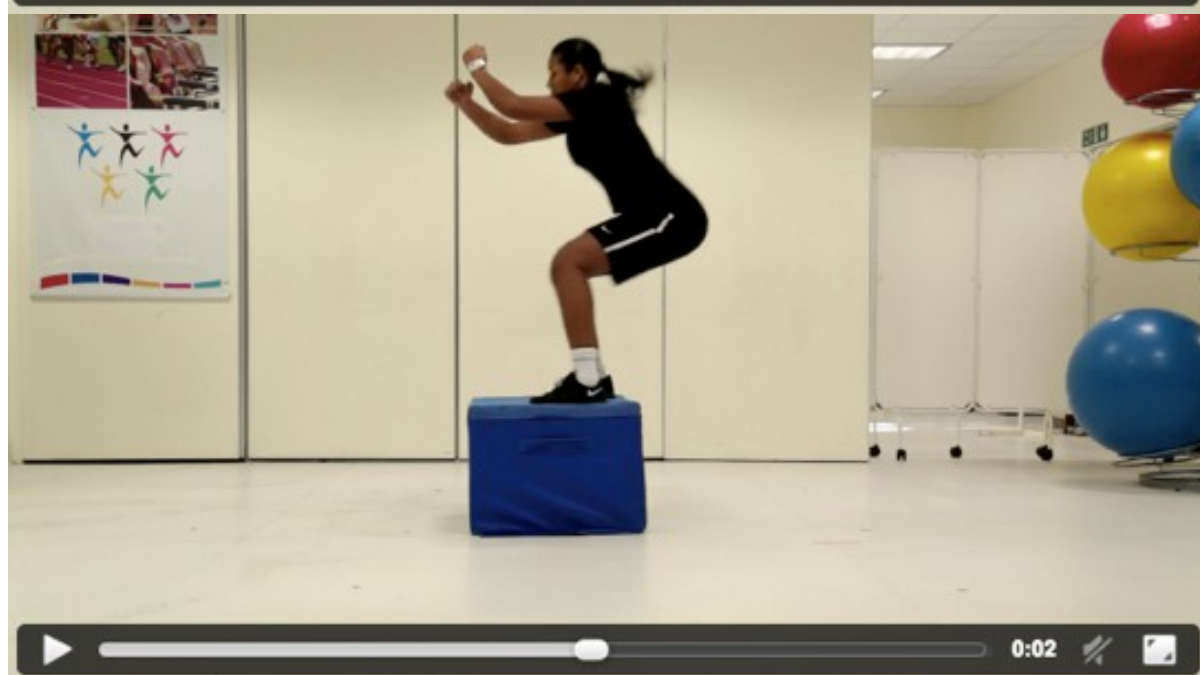

Supplement: Multimedia Appendix 1 [file resprot_v5i4e234_app1.pdf]
